# The Effects of a Mindfulness-Based Training in an Elementary School in Germany

**DOI:** 10.1007/s12671-023-02084-w

**Published:** 2023-02-07

**Authors:** Christiane Portele, Petra Jansen

**Affiliations:** Faculty of Human Science, University of Regensburg, Universitätsstraße 31, 93053 Regensburg, Germany

**Keywords:** Mindfulness, Emotion regulation, Primary school, Elementary school, Education system

## Abstract

**Objectives:**

The primary goal of this study was to investigate the influence of the Mindfulness Education Workbook, a 6-week mindfulness-based tool, on emotion regulation, inhibition, physical self-concept, resources, and connectedness to nature. Furthermore, we explored whether a difference in number of hours of mindfulness practice would affect the outcomes.

**Method:**

Ninety-one children from a public elementary school (*M* age = 9.74 years, *SD* = 0.76) participated in the study and were divided into three groups according to their respective school classes. The intervention group was divided into two groups that varied by number of hours of mindfulness practice: (a) mindfulness-plus and (b) mindfulness. In addition to biweekly training, the mindfulness-plus group also repeated a daily exercise. The passive control group received the standard school day instruction. The five concepts of emotion regulation, inhibition, physical self-concept, resources, and connectedness to nature were measured before and after the mindfulness intervention.

**Results:**

For the measurement of emotion regulation, there was a significant effect in favor of the two mindfulness groups compared with the control group for the adaptive strategies in total as well as for their comprising emotions, anger, fear, and sadness, separately. Solely for the subscale *mood elevation*, the mindfulness-plus group showed significantly higher scores compared to the control group. Both mindfulness-plus and mindfulness groups varied from the control group on the measure of emotion regulation strategies, however not on the other four domains that were assessed (self-reports of resources, physical self-concept, and connectedness to nature as well as a mental task assessing inhibition). There was no evidence that the additional practice in the mindfulness-plus group significantly added to the intervention’s effectiveness.

**Conclusions:**

The Mindfulness Education Workbook is a promising tool for elementary schools. Follow-up studies may provide further insights into the various effects of offering mindfulness training in schools. Further research with objective markers may also allow individual aspects under the umbrella term mindfulness to be investigated in more detail.

**Preregistration:**

This study was not preregistered.

**Supplementary Information:**

The online version contains supplementary material available at 10.1007/s12671-023-02084-w.

The fast-changing world with its accumulating challenges demands that educational systems change appropriately to equip young and upcoming generations with the tools necessary to not only face those challenges but to also live well and be healthy. Combining and balancing heart, mind, and body to cultivate wisdom, compassion, and well-being in addition to intelligence is essential to thinking in more naturalistic, holistic, and integrative ways (Hawkins & Burke, 2021), which should be a goal of education. Mindfulness, as an awareness of being in the present moment nonjudgmentally (Kabat-Zinn, 2003), is able to achieve the described balance. The mechanisms of mindfulness are attention regulation (e.g., sustaining attention on the chosen object), body awareness (the sensory experience of breathing, emotions, or other body sensations), emotion regulation (which includes the components of reappraisal; exposure, extinction, or reconsolidation), and a change in perspective (detachment from identification with a static sense of the self) (Hölzel et al., 2011).

Many programs that incorporate the concepts of mindfulness, mindfulness-based awareness, and social and emotional learning, or similar constructs, have been developed for the educational context. Despite their diversity, these programs share an underlying commonality: supporting children in cultivating a sense of well-being so they can thrive and flourish in the daily hustle (e.g., Schonert-Reichl et al., 2015). To date, several programs for elementary school–age children have been developed, for example, Mindfulness-Oriented Meditation for primary school children (Crescentini et al., 2016), a Still Quiet Place (Saltzman, 2014), Paws b (ages 7–11), the Mindfulness in Schools Project (Vickery & Dorjee, 2016), MindUP (Hai et al., 2021), the Mindful Education Workbook (Rechtschaffen, 2016), Learning to BREATHE for K–12 (Broderick, 2021), the Inner Kids Program developed by Susan Kaiser Greenland (Flook et al., 2010), CalmSpace (Janz et al., 2019), the Living Mindfully Programme (Amundsen et al., 2020), and the Gaia Program (Ghiroldi et al., 2020).

According to the mechanisms described by Hölzel et al. (2011), mindfulness training might influence emotional, cognitive (attentional), bodily, and perspective-changing abilities. Regarding emotional abilities, the practice of mindfulness was associated with healthy emotion regulation in one study (Roemer et al., 2015); however, another study did not find a significant improvement in emotion regulation (Amundsen et al., 2020). Amundsen et al. (2020) examined the effectiveness of a 6-week mindfulness program (Living Mindfully Programme, UK) for 9- to 10-year-old children on outcomes such as well-being and emotion regulation. Schoolteachers delivered the program as part of their school curriculum. Compared with the wait-list control group, children in the mindfulness group improved significantly in mindfulness, positive outlook, and life satisfaction immediately after the intervention and at a 3-month follow-up. Compared with the active control group, which received a quasi-positive psychology intervention, the effects remained, however, in a reduced form.

A further study examined an 8-week mindfulness program (Paws b) for children ages 7 to 9 years offered by schoolteachers within a regular school curriculum (Vickery & Dorjee, 2016). The mindfulness groups demonstrated a decrease in negative affect with a large effect size at follow-up. In another study, the effectiveness of an elementary school–based mindfulness intervention for 8- to 12-year-old children was assessed (van de Weijer-Bergsma et al., 2012). Children were randomly assigned to an intervention or a wait-list control group. Children receiving the 6-week MindfulKids training showed only a few primary prevention effects on stress and psychological well-being directly after the intervention. At follow-up (7 weeks after posttesting), more effects were found. Compared with the pretest, there was a significant increase in child-reported differentiation of emotions, verbal sharing of emotions, bodily awareness, not hiding emotions, and sense of coherence, and a significant decrease in rumination and analyzing emotions. Furthermore, anxiety and angry/aggressive behavior reported by parents declined significantly.

Schonert-Reichl et al. (2015) investigated the effectiveness of MindUP (a social and emotional learning program with mindfulness practices) on stress regulation, social-emotional competence, and school achievement in children age 9 to 11 years. The program focuses on promoting self-regulation, social-emotional understanding, positive mood (learning optimism, practicing gratitude), and acts of kindness, among other aspects. The results were compared with those of an active control group who took part in a business-as-usual (BAU) social responsibility program. Compared with the business-as-usual group, the MindUP group showed significant improvement in self-report measures of well-being and self- and peer-reported prosocial behavior. In another study, children aged 7 and 8 years who completed 8-week training in either a mindfulness-orientated meditation or an emotion awareness training were both rated by their schoolteachers as displaying reduced internalizing emotional problems (Crescentini et al., 2016).

With regard to cognitive processes, a systematic review described the generally positive impact of mindfulness-based interventions (MBIs) on executive functions (EFs) in studies published until 2015 (Jansen et al., 2016). EFs are essential cognitive control functions, namely, (a) working memory, conceptualized as *updating* and monitoring working memory processes; (b) cognitive flexibility, which involves a rapid switching or *shifting* between tasks and thought processes; and (c) *inhibition*, the ability to react to relevant stimuli and to suppress irrelevant stimuli (Miyake et al., 2000). The results of the review have been confirmed by further studies, for example with improvements in 6-year-old elementary school children in cognitive flexibility and inhibition skills after the mindfulness intervention CalmSpace compared with a wait-list control group (Janz et al., 2019). This result was confirmed by teacher reports.

As of now, no study has investigated the influence of mindfulness training on bodily-based processes, such as interoception. In one pilot study, the effect of yoga training was investigated with regard to the physical self-concept (Richter et al., 2016), including the perception of one’s body. Yoga can be described as a mindful form of movement (Jansen et al., 2019). In contrast to the physical skills in the training group, that showed an increase in the mean perceived speed in the physical self-concept questionnaire, the yoga group showed a decrease. The physical self-concept is relevant because it is related to general self-concept and mathematical achievement in primary school–age children with compensatory education needs (Del Palomino, 2017).

Regarding the fourth mechanism perspective-changing (Hölzel et al., 2011), no study has yet investigated this in primary school–age children who have completed an MBI. However, when a broader interpretation is made, changing perspective can be conceptualized as the ability to connect with others or the environment. Jalón et al. (2022) investigated mindful, empathic, and pro-environmental attitudes in elementary school pupils aged 10 to 12 years after an MBI training that was delivered in 5-min practice sessions three times per week over a course of 4 months, by the schoolteacher who had experience with meditation. Compared with the passive control group, the MBI group improved significantly, with large effects on mindfulness skills and pro-environmental attitudes and medium effects on considerate social style and cognitive empathy. In conclusion, almost all of the concepts highlighted by Hölzel et al. (2011) have been addressed as outcomes for mindfulness interventions with school-aged children in other studies, but so far not in one single study, that included the concept of connectedness to nature. In general, we assume that increased practice and repetition strengthen the effects of MBIs (Flook et al., 2010; Lillard, 2011).

In the present study, we aimed to identify the effect of a mindfulness training on elementary school–age children to examine the different mechanisms through which mindfulness functions (Hölzel et al., 2011). To satisfy the requirement of the current state of research to investigate mindfulness with its subfacets and the targeted effects of the components of mindfulness, we aimed to find tests for each component and added the concept of connectedness to nature. In an exploratory manner, we established a mindfulness-plus group to investigate whether a higher amount of practice (mindfulness-plus) positively supports the effects of the training. We hypothesized that, compared with a control group, the mindfulness-plus and the mindfulness groups would improve in the domains of emotion regulation, resources, attentional processes, bodily based processes, and connectedness to nature. Finally, we hypothesized that the mindfulness-plus group would perform better compared to the mindfulness group on the five measured outcomes.

## Method

### Participants

We conducted a power analysis with G*Power (Faul et al., 2007) and, assuming a value between a low and medium effect size *d* = 0.175, an alpha level of *p* = 0.05, and a power of *1 − ß* = 0.8, a sample size of 84 was calculated. Ninety-three children were recruited from three third grade and three fourth grade classes of a state elementary school with an external site in a city in southeast Germany. The parents of two children withdrew their consent for them to participate in the study after they were asked to complete the socioeconomic status questionnaire. Therefore, the final sample size was 91 (*M* age = 9.74 years, *SD* = 0.76, range: 8–11). The six school classes were assigned to three different conditions (mindfulness-plus, mindfulness, and control) with two classes assigned to each condition.

Children in the mindfulness-plus group (*n* = 32; 18 girls and 14 boys; *M* age = 9.72 years, *SD* = 0.89) received the mindfulness training, and in addition to this their schoolteachers repeated a mindfulness exercise with them each day in school. The two classes allocated to mindfulness-plus were assigned to this condition because the schoolteachers of these classes had experience with mindfulness meditation. The remaining classrooms were randomly assigned to the other two conditions. In the mindfulness group (*n* = 31; 19 girls and 12 boys; *M* age = 9.55 years, *SD* = 0.81) children participated in the mindfulness training but did not complete a daily mindfulness exercise, and children in the control group (*n* = 28; 17 girls and 11 boys; *M* age = 9.96 years, *SD* = 0.43) followed the normal school routine. At the end of the project, the control group received a sample session to experience mindfulness exercises.

In accordance with Lampert et al. (2018), the socioeconomic status (SES) index was generated as a household characteristic on the basis of parental information on the three scales: Education, occupation, and income. Some respondents generally do not provide information on their income situation (Riphahn & Serfling, 2005). In this study, 40 SES questionnaires were completed fully, 17 SES questionnaires were not filled out, and 34 parents did not specify their incomes but completed the rest of the questionnaire. These data were thus imputed from education and occupation data and income values from other parents who had the same or similar education and occupation scores.

### Procedure

The 6-week mindfulness intervention was embedded in 1 week of pre-and posttesting. The mindfulness lessons were conducted twice per week for 1 school hour (45 min) in the classrooms of the two mindfulness-plus and the two mindfulness groups. Both groups received handouts for each lesson with exercises and small tasks to voluntarily do at home. In the mindfulness-plus groups only, the schoolteachers were asked to complete a daily mindfulness exercise by choosing any exercise from the curriculum. The schoolteachers were free to choose any exercise and were not given any specific instructions on how long the daily exercise sessions should last. Additionally, schoolteachers were told that the children themselves could lead daily exercises once they had become comfortable with the program. In the two mindfulness-plus groups, the training was conducted in a circle of chairs; in all other groups, the children sat at their school desks.

The four questionnaires were administered in a group testing format in class with the schoolteacher. Before the start of the first test, the schoolteacher read an instruction explaining that there are no right or wrong answers and that answers should be given as spontaneously as possible. Questions about terminology and answer scales were given according to the recommendations in the test manuals. The individual tests were performed at time intervals to avoid fatigue effects. The Flanker task took place in a one-to-one setting with a transparent partition between the experimenter and the child for COVID-19 infection control reasons. In addition to the written instructions on the computer, oral instructions were given uniformly. A cover story for children was used to improve attention and commitment during testing: Using the two arrow keys, they were asked to feed a hungry fish in the middle that was flanked by two others. Upon completion of the task, candy was given as a reward.

The mindfulness lessons were adapted from the Mindful Education Workbook (Rechtschaffen, 2016, 2018). Rechtschaffen (2016) built his lessons according to different realms and emphasized that mindfulness skills develop best through progressive stages. The exercises are assigned to the five concepts: (a) physical, (b) mental, (c) emotional, (d) social, and (e) global. The *physical concept* is about the language of the body, being present and regulated in one’s own body. The *mental concept* deals with mechanics of the mind, witnessing thought patterns, and developing focusing skills. The basis for the *emotional concept* includes regulating difficult emotions and enhancing good feelings. The next step, the *social concept*, brings the learned skills into social dynamics, communicating compassionately and listening deeply. The *global concept* is about interconnectedness with everything in the world.

Teaching children mindfulness in a fun way, quasi –‘playing mindfulness’ is one of the main foci of the program. Breathing exercises in which breathing patterns are combined with animal or nature movements, popcorn thoughts (one half of the class always raises the hand when a thought occurs, the other half tries to count the number of thoughts), heartful phrases (a sort of loving kindness mediation), rose and thorn (one child tells a good story that happened to them (rose) and then about a not so good story (thorn), another child listens deeply and sums up the rose and thorn stories; subsequently, narrator and listener roles are swapped), and life cycle assessment, represent one sample exercise from each concept. There were two lessons per concept, so the children participated in a total of 10 mindfulness lessons, twice per week, for 45 min each. An eleventh lesson was used to provide integration, review, recap, and time for favorite exercises (for an overview, see Table 1). The lessons were team-taught by an external mindfulness-based stress reduction (MBSR) trainer experienced in elementary education and trained by Daniel Rechtschaffen as a Mindful Education Teacher Trainer together with an assistant trained in mindfulness with children. Because of the COVID-19 pandemic, hygiene, and infection protection measures, such as face masks, regular ventilation, and so on, were implemented depending on the current number of infections and the requirements of the school.

**Table 1:** Overview of the lessons based on the Mindful Education Workbook (Rechtschaffen, 2016)

The five realms of mindful concepts	Lessons 1–11
Physical	Lesson 1 Exploring the language of the body Lesson 2 Moving and exploring the breath
Mental	Lesson 3 Throwing an anchor – breathing and listening Lesson 4 Throwing an anchor – seeing and thinking
Emotional	Lesson 5 Being happy and making happy Lesson 6 Stressful feelings and their roots
Social	Lesson 7 Inner and outer weather report and nonverbal communication Lesson 8 Mindful communication
Global	Lesson 9 Mindful eating and knowing your world Lesson 10 Integration practices
	Additional Lesson 11 Repetition and favorite exercises

### Measures

To assess emotional processes, the questionnaire for the assessment of emotion regulation in children and adolescents [Fragebogen zur Erhebung der Emotionsregulation bei Kindern und Jugendlichen] (FEEL-KJ; Grob & Smolenski, 2005) was applied. This instrument measures multidimensional and emotion-specific emotion regulation strategies for the three emotions of anger, fear, and sadness. We looked at seven adaptive strategies (problem-focused action, distraction, mood elevation, accepting, forgetting, cognitive problem solving, and reevaluating), with 42 items; five maladaptive strategies (giving up, aggressive behavior, withdrawal, self-deprecation, and perseveration), with 30 items; and three other strategies that could not be assigned to any of the other two secondary scales (expression, social support, and emotion control), with 18 items. There are two items for each of the 15 strategy options and each of the strategy options is measured for each of the three emotions, resulting in 90 items in total (Goldschmidt & Berth, 2006). The answer format consists of a 5-point scale that ranges from 1 (*almost never*) to 5 (*almost always*).

The test manual’s internal consistency was satisfactory, with a Cronbach’s *α* across emotions between 0.69 (giving up, forgetting, and perseveration) and 0.91 (social support). In the present study, the internal consistencies in the pretest were very good (Cronbach’s *α* = 0.94, McDonald’s *ω* = 0.93, for 41 items) for the adaptive strategies, with good reliabilities for each of the adaptive strategies for anger (Cronbach’s *α* = 0.85, McDonald’s *ω* = 0.85), fear (Cronbach’s *α* = 0.85, McDonald’s *ω* = 0.84), and sadness (Cronbach’s *α* = 0.88, McDonald’s *ω* = 0.88). The internal consistency for the maladaptive strategies was satisfactory (Cronbach’s *α* = 0.81, McDonald’s *ω* = 0.76). The internal consistencies for the three other strategies were satisfactory for expression (Cronbach’s *α* = 0.71, McDonald’s *ω* = 0.65) and good for social support (Cronbach’s *α* =0.81, McDonald’s *ω* = 0.81) but not for emotion control (Cronbach’s *α* = 0.61, McDonald’s *ω* = 0.59).

The Flanker task (Eriksen & Eriksen, 1974) measures inhibitory control, one of the three main components of EFs. The inhibition tasks require attentional processes and are therefore adequate to measure the attentional mechanism of mindfulness. The description of the task is presented in supplementary material.

The physical self-concept questionnaire for elementary school–age children [Fragebogen zur Erfassung des physischen Selbstkonzepts von Kindern im Grundschulalter] (PSK-K; Dreiskämper et al., 2015) was used. The PSK-K contains 21 items in total, consisting of seven scales (endurance, flexibility, strength, coordination, speed, global sport competence, and physical appearance). Each scale has three associated questions and a 4-point scale response format that ranges from 1 (*not at all*) to 4 (*absolutely*). Higher total scores indicate higher levels of self-concept. The internal consistency of the seven scales in the test manual (Cronbach’s *α*) ranges between 0.57 and 0.82. The internal consistency of the seven scales in our study varied between 0.61 and 0.87 for both Cronbach’s *α* and McDonald’s *ω*.

The questionnaire that addressed resources in childhood and adolescence [Fragebogen zu Ressourcen im Kindes- und Jugendalter] (FRKJ 8-16; Lohaus & Nussbeck, 2016) was conducted in the realm of social abilities. The FRKJ 8-16 can be used to measure developmental resources in children who have been distinguished regarding available personal development and environmental resources. Empathy and perspective-taking skills, self-efficacy, self-esteem, a sense of coherence, optimism, and self-control are the constructs measured for personal development resources. For the environmental resources, parental support, authoritative parenting style, peer group integration, and school integration are the measured constructs. The answer format consists of a 4-point scale that ranges from 1 (*never true*) to 4 (*always true*). Each questionnaire scale consists of six items, resulting in 60 items. One item is reverse coded. A high numerical score indicates a high resource level. Cronbach’s *α* for the internal consistency for the 10 scales in the test manual varies between 0.69 and 0.89 and, in our study, between 0.63 and 0.89 (Cronbach’s *α*) and between 0.62 and 0.89 (McDonald’s *ω*).

To measure more global processes, the questionnaire on connectedness to nature (Otto & Pensini, 2017), a 20-item shortened version of the Disposition to Connect to Nature Scale (Brügger et al., 2011) was used. The scale is suitable for children. The response format is a 5-point scale that ranges from 1 (*not at all*) to 5 (*absolutely*). “I feel the need to be out in nature” and “Watching animals is exciting” represent two example items from the scale. Two items are reverse coded. A total score is formed from all 20 items: The higher the total score, the higher the connectedness to nature. The scales are based on a Rasch model.

### Data Analyses

According to the instructions of the FEEL-KJ test manual, if an item response is missing, the mean value is calculated from the available five out of six answers and rounded to an integer. For the FRKJ 8-16, the test authors also recommended that if only one item is missing, the arithmetic mean of the remaining five responses on the scale should be substituted. In the data preparation, trials in the Flanker task with reaction times lower than a cutoff value of 200 ms were first omitted. Following the recommendations of Baayen and Milin (2010), trials with reaction times 2 *SD* below or above a participant’s mean reaction time were also excluded. Because of the given feedback after each trial, error trials and hits after an error were sorted out for the reaction times. In accordance with the suggestions by Richter et al. (2016), participants with reaction time values 1.5 times the interquartile range above or below the sample median were excluded from individual analyses for the Flanker task. The exclusion was done separately for the congruent and incongruent conditions and pre- and posttests. For posttest data, outliers were defined for each group individually because of possible group differences after the mindfulness intervention. Further exclusion reasons or missing data (e.g., if a participant was not present at posttesting) are stated in the “Results” section for each test.

Before further calculations, we conducted one-way analyses of variance (ANOVAs) to determine whether the three groups differed in age and SES, and we used a *χ*^2^ test to detect possible gender differences. We then performed repeated-measures ANOVAs, with the total scores of the FEEL-KJ, FRKJ 8-16, PSK-K, and connectedness to nature questionnaire; the subscale scores of the FEEL-KJ and the FRKJ 8-16; and reaction times and hit rates in the Flanker task as dependent variables. The time of measurement (pre, post) serves as a within-subject factor, and the three groups (mindfulness-plus, mindfulness, control) as between-subjects factors. For the Flanker task, another within-subject factor condition (congruent, incongruent) was added for a three-way analysis. If interaction effects were significant, paired *t*-tests were computed. The significance level for all analyses was set to *p* = 0.05. If there was a significant effect on the factor group, two separate ANOVAs were calculated for the posttest scores. In this case, the significance level for all analyses was Bonferroni corrected and set to *p* = 0.025.

## Results

One-way ANOVAs showed that there was no significant difference between the three groups (mindfulness-plus, mindfulness, control) regarding age, *F*(2, 88) = 2.29, *p* = 0.107, *η*_p_^2^ = 0.050, and SES, *F*(2, 71) = 0.22, *p* = 0.804, *η*_p_^2^ = 0.006. Because 17 parents had not completed the SES questionnaire, the sample size for the SES calculations was *n* = 74 (mindfulness-plus: *n* = 27, mindfulness: *n* = 26, control: *n* = 21). The *χ*^2^ test showed that the number of boys and girls did not differ between groups, *χ*^2^(2) = 0.197, *p* = 0.906, *φ* = 0.047. Due to no differences in the pre-test, age, gender, and SES were not considered in the following statistical analyses.

### Emotion Regulation (FEEL-KJ)

For the total score of the strategies and the subscales, the sample was *n* = 90 because one child in the mindfulness group had not completed more than one item in each strategy, and thus the mean could not be substituted. Repeated-measures ANOVA results with all main and interaction effects for the adaptive strategies of the FEEL-KJ are shown in Table 2. In all pretests, there was no difference between groups (all *p >* 0.205). The last column shows the differences (Bonferroni-corrected) between the groups in the posttests. For most of the adaptive strategies, there were statistically significant interaction effects of group × time (*p* < 0.05) with a better performance in the mindfulness group(s).

**Table 2:** Repeated-measures ANOVAs for differences between the three groups (mindfulness-plus (M+), mindfulness (M), and control group (CG)) in the adaptive strategies in total, for anger, fear, and sadness of the FEEL-KJ

Dependent variable	Main and interaction effects	*n*	*F* (*df*_eff_,*df*_err_)	*p*	*η*_p_^2^	Differences between M+, M, and CG
FEEL-KJ – AD-S						
Total		90				
	Effect of time Effect of group Time × group		0.21 (1,87) 3.02 (2,87) 2.33 (2,87)	0.649 0.054 < 0.05	0.002 0.065 0.129	M+ > CG; M > CG
*ANG*		90				
	Effect of time Effect of group Time × group		0.03 (1,87) 2.90 (2,87) 4.20 (2,87)	0.867 0.063 < 0.05	< 0.001 0.062 0.125	M+ > CG; M > CG
*FEA*		90				
	Effect of time Effect of group Time × group		0.46 (1,87) 3.20 (2,87) 0.24 (2,87)	0.502 < 0.05 < 0.05	0.005 0.068 0.088	M+ > CG; M > CG
*SAD*		90				
	Effect of time Effect of group Time × group		0.11 (1,87) 2.21 (2,87) 3.22 (2,87)	0.738 0.115 < 0.05	0.001 0.048 0.069	M > CG; M+ = CG; M+ = M

*AD-S*, adaptive strategies; *ANG*, anger; *FEA*, fear; *SAD*, sadness

All means and standard deviations for the variables and for the three groups in pre- and post-testing are listed in Table 3.

**Table 3:** Pre- and post-test means and standard deviations for significant variables of the FEEL-KJ

Dependent variable	Control		Mindfulness		Mindfulness-plus	
	Pre	Post	Pre	Post	Pre	Post
	*M* (*SD*)	*M* (*SD*)	*M* (*SD*)	*M* (*SD*)	*M* (*SD*)	*M* (*SD*)
FEEL-KJ – AD-S						
Total	126.29 (33.01)	114.29 (37.26)	134.53 (25.75)	142.13 (32.38)	132.00 (33.67)	139.91 (33.24)
*FEA*	41.68 (13.00)	38.18 (14.07)	44.70 (10.24)	47.70 (11.86)	45.13 (11.16)	47.66 (11.89)
*ANG*	42.11 (11.55)	37.00 (12.86)	45.37 (9.02)	46.20 (12.11)	42.72 (12.22)	46.47 (11.48)
*SAD*	42.50 (13.04)	39.14 (12.28)	45.20 (9.43)	48.07 (11.02)	44.16 (12.72)	45.69 (12.11)

*AD-S*, adaptive strategies; *FEA*, fear; *ANG*, anger; *SAD*, sadness

The results for the maladaptive strategies and other strategies are presented in the supplementary material.

### Inhibition (Flanker Task)

After the exclusion of outliers, *n* = 75 (mindfulness-plus: *n* = 27, mindfulness: *n* = 25, control: *n* = 23) remained for the following analyses. The main effect of condition was significant, *F*(1, 72) = 16.75, *p* < 0.001, *η*_p_^2^ = 0.189, confirming the Flanker effect with shorter reaction time for the congruent (*M* = 623.85, *SD* = 117.25) compared to the incongruent (*M* = 641.79, *SD* = 124.39) items. Regarding reaction time, the three-way interaction between time, condition, and group was not statistically significant, *F*(2, 72) = 0.733, *p* = 0.484, *η*_p_^2^ = 0.020.

Secondly, the main and all possible interaction effects of group, time, and condition in the repeated-measures ANOVA for the hit rate were not statistically significant (all *p* > 0.051).

### Physical Self-Concept (PSK-K)

Here, the sample was *n* = 90 because one child in the control group did not have posttesting results. The repeated-measures ANOVA for the difference in the overall PSK-K score between the groups after the intervention revealed only a significant effect of time, *F*(1, 87) = 10.57, *p* = 0.002, *η*_p_^2^ = 0.108.

### Resources (FRKJ 8-16)

The sample in the total score and for the subscales varied between *n* = 88 and 90. One child was not present at posttesting, and two others had too few questions completed in some subscales to form a score. Results for the repeated-measures ANOVAs are listed in Table S1. Only the subscale parental support showed a significant main effect of group and interaction effect of time × group. There was a difference in the pretest between groups, *F*(2, 88) = 6.56, *p* = 0.002, *η*_p_^2^ = 0.130, and no difference in the posttest, *F*(2, 85) = 1.39, *p* = 0.254, *η*_p_^2^ = 0.032. Bonferroni-corrected post hoc tests showed that in the pretest, the control group (*M* = 22.62, *SD* = 2.04) showed significant higher values compared to the mindfulness-plus group (*M* = 19.91, *SD* = 4.45; *p* = 0.010) and the mindfulness group (*M* = 19.67, *SD* = 3.07; *p* = 0.004). The mindfulness-plus and mindfulness group did not differ significantly (*p* = 1.000). Some other subscales reached a significant main effect of time but there was no significant difference in terms of an increase in measured resources in favor of the mindfulness groups.

### Connectedness to Nature

As with the PSK-K, the sample was *n* = 90 because one child in the control group did not have posttesting results. There were no significant effects at all (all *p* > 0.05).

## Discussion

First, this study demonstrates a promising practical implementation of an externally delivered MBI in an elementary school. A strength of this study is the examination of several concepts theorized to have a connection to mindfulness. Our study provides evidence that mindfulness training in children influences emotional processes. However, in this sample, there was little support for the other four concepts. Furthermore, it showed that the frequency of mindfulness exercises did not affect the outcome.

Regarding emotion regulation, most of the adaptive strategies improved in favor of the two mindfulness groups compared with the control group. Both mindfulness groups scored significantly higher compared to the control group on all but one of the subscales; however, the two mindfulness groups did not differ from each other as hypothesized. Only for the mood elevation subscale did the mindfulness-plus group outperform the mindfulness group with the children in this group scoring significantly higher compared to the children in the mindfulness and control groups which did not differ significantly from each other. The results showed that despite a higher level of exposure to mindfulness practice in the form of daily exercises, the mindfulness-plus group condition produced an almost equal increase in emotion regulation scores. This brings into question the benefit of the daily exercises and suggests that they did not have as much of an impact as the biweekly sessions led by the mindfulness trainers. It is not clear why the daily exercises did not contribute to a wider increase in emotion regulation. One possible explanation could relate to the implementation of the daily exercises. Schoolteachers were not given specific instructions of how long the sessions should be or which particular exercises they should focus on and it may be that the sessions were too short. Future studies investigating the most effective dose of mindfulness training could use some form of implementation measure for example a diary where schoolteachers can record the length of the sessions, and which exercise was chosen.

During the course of the program, we observed that allowing the children to lead mindfulness exercises appeared to represent an enriching addition. Regarding the dose of mindfulness training, one meta-analysis of children and adolescents showed that more mindfulness training reduced negative behavior in children and adolescents (Dunning et al., 2019). In contrast, fewer hours of mindfulness training resulted in a better outcome in well-being in a study conducted by Dunning et al., 2022. The results of our study do not align neatly with either of these past studies, instead suggesting that an increase of mindfulness training does not produce any major additional positive nor negative results with some small additional benefits in elevated mood.

For Rechtschaffen (2016), the concept of emotion regulation relates in particular to regulating difficult emotions and enhancing good feelings. The emotion lessons focused on good wishes for the self and others, gratitude, and anonymous good deeds for others. In addition, the children learned some exercises about how to better learn to surf emotional waves and emergency exercises, like ‘vacuum cleaner breaths’, for very strong, overwhelming feelings. The exercise involves taking a deep breath, holding it for a moment and then letting the breath out with a sound, three times maximum. Although the emotional concept section only constituted two lessons within the curriculum, it may be that the children’s emotion regulation improved most because of how the program was implemented. The two mindfulness trainers exemplified a very empathic approach to the children. This empathic approach is an underlying foundation of the program and was not only restricted to the emotional concept lessons. These results align with those of several studies (e.g., Amundsen et al., 2020) in which emotional and behavioral problems improved after MBIs. The outcomes of the self-completion surveys and teachers’ reflections in the study by Joyce et al. (2010) indicated improvements in emotional health, in particular for children who scored in the borderline and abnormal categories before the MBI. In another study, fifth-grade pupils showed significant improvements in most areas of mental health (emotions, behavior, relationships, and prosocial behavior) and quality of life scores (Waldemar et al., 2016).

Regarding attentional and cognitive processes, the results showed that the mindfulness-plus and mindfulness groups differed significantly in reaction times from pre- to post-test, but the control group did not. However, this result was independent of the congruency of the stimuli, meaning that no significantly improved inhibition ability was demonstrated relative to the control group. To further explore the possible influence of mindfulness programs on attentional tasks, future studies should investigate attentional tasks beyond inhibition. The hit rate in the task was generally very high, which may suggest a ceiling effect and that the task could possibly have been too easy for elementary school students. These findings are consistent with those of Richter et al. (2016), who also found generally low error rates. This repeated result suggests that future studies with older elementary school children should explore using a version of the Flanker task which has been adapted to be more challenging, possibly by increasing the number of stimuli being presented during a trial.

To further explore the possible influence of mindfulness programs on attentional tasks, future studies should investigate attentional tasks beyond inhibition. Butterfield and Roberts (2022) discussed the role of EFs in children’s experiences of mindfulness because EFs may also matter for understanding nonsignificant outcomes in MBIs. The two authors consider that research on MBIs often presents nonsignificant results without appreciating the significant benefits that might accrue for a subset of participants. The duration of the MindfulMe! mindfulness training also lasted 6 weeks and was conducted by external trainers.

None of the three measurements of bodily based, social, and global processes showed significant improvement. For the measurement of bodily processes, it may be that the PSK-K focuses too much on the athletic component, and it may be better to use test measurements (e.g., the heartbeat perception task for interoception and self-perception) in future studies. The physical element of mindfulness refers more to a sense of inner satisfaction or a friendly, compassionate attitude toward one’s own body than to components such as endurance, speed, and physical appearance. For social processes, it would be useful to find other tests that increasingly measure the social component, for example, in the study by Flook et al. (2015) of the measurement of prosocial behavior.

There have been few studies with elementary school-age children that measure resources. When studies have addressed the topic of resources, they have tended to measure subjective well-being, which is generally strongly associated with personal and social resources (Diener & Fujita, 1995), such as the subjective well-being measured by the Student’s Life Satisfaction Scale and the Sterling Children’s Wellbeing Scale (Amundsen et al., 2020). The results showed that, compared with the wait-list or active control group, the mindfulness training significantly enhanced self-reported domains of well-being-positive outlook and life satisfaction-with medium and low effect sizes, respectively. Given that the children were not out in nature during the lessons but instead focused on sounds and theoretical considerations of the interconnectedness of the world and on life cycles, it could be that these possible small changes cannot yet be measured in a test that is purely specialized for connectedness to nature. There exists only one study that measured environmental attitudes, with the New Ecological Paradigm Scale for Children. After that MBI, significant improvements in pro-environmental attitudes with large effects were found (Jalón et al., 2022).

### Limitations and Future Research

Several limitations of this study should be noted. One methodological limitation was that children could not be randomly allocated to the intervention or control groups. The mindfulness and control groups consisted of already-existing homeroom classes. This is due, on the one hand, to the nature of field studies and the required adaptation of the study’s procedures to the specifications of the schools and, on the other hand, to the pandemic situation, during which an exchange between the pupils of the existing school classes would not have been feasible. However, in school-based research of mindfulness, the cluster randomization due to the existing social group is likely part of its effects.

Another limitation is that the entire project took place during the COVID-19 pandemic. Whether this fact affects differences in the results is difficult to judge retrospectively. Cullen et al. (2020) urged the development of measures to mitigate the negative effects of the pandemic. Mindfulness meditation training has been suggested as one promising intervention (Yuan, 2021). Thus, integrating mindfulness interventions into the school day could be a valuable measure to promote mental health in pandemic times (Butterfield & Roberts, 2022).

Moreover, in addition to the passive control group, it would be useful to include an active control group (e.g., with relaxation techniques) in future studies to increase internal validity (Jalón et al., 2022). Butterfield and Roberts (2022) assumed an overestimation of the positive effects due to mindfulness compared with a passive control group who took part in their normal school routine. In addition, because this study involved fourth graders who moved on to secondary school after the school year, follow-up testing was not possible for organizational reasons. Six months after the mindfulness training, teachers reported that the pupils continued to engage in mindfulness practices, suggesting a sustainable implementation to explore in future projects. Follow-up testing and studies could provide further important insights. In several studies, even larger effects have been obtained in follow-up measurements (Amundsen et al., 2020; van de Weijer-Bergsma et al., 2012; Vickery & Dorjee, 2016).

To create optimal learning environments in schools, involving the entire school family in the topic of mindfulness would be desirable. For a sustainable, long-term implementation, the schoolteachers should be involved in the training and not only participate but also train themselves in mindfulness initially as a means of teaching mindfully and, later, to teach mindfulness. Hawkins and Burke (2021) suggested three levels for successfully integrating mindfulness in the education system: (a) being mindful, (b) teaching mindfully, and (c) teaching mindfulness. In no case should schoolteachers have to teach mindfulness if they themselves do not stand behind the concept. A joint introductory event with practical content for the entire school community is recommended because everyone gets the same insight, and prejudices can be counteracted.

## Supplementary Information

ESM 1: Table S1 (DOCX 19.5 kb)

## Data Availability

Data can be obtained from the corresponding author upon an email request.
