# Supplementary material for: The Effects of a Mindfulness-Based Training in an Elementary School in Germany
Source: Mindfulness (N Y). 2023 Feb 7;14(4):830–40. doi: 10.1007/s12671-023-02084-w (PMC9902837; doi:10.1007/s12671-023-02084-w)
Supplement: Supplementary file 1 — Table S1 (DOCX 19.5 kb) [file 12671_2023_2084_MOESM1_ESM.docx]

**Supplementary Material**

**Description of the Flanker Task**

For this task, the child sat in front of a 15-inch laptop. First, a black fixation cross was presented for 2000 ms on a white screen at the beginning of the experiment. All further stimuli appeared without a fixation cross. Afterward, three bluish fish were presented until a key press occurred. The child’s task was to react as quickly as possible to only the hungry fish in the middle. The right or left arrow key should be pressed depending on whether the fish in the middle is pointing to the right or left side. One of the four conditions appeared randomized: In the two congruent conditions, all three fish are looking either on the left or right side, while in the two incongruent conditions, the two flanked fish point in the opposite direction compared to the target stimulus in the center. Therefore, reaction times are expected to be longer due to distraction by the two flanked fish. Ten trials per condition resulted in 40 trials in total. Either a yellow smiley with a happy face after a correct keystroke or a yellow smiley with a sad face after an incorrect keystroke was presented for 2500 ms. The child was instructed to continue normally even after a sad faced smiley. At the end of the test, a smiley with a happy face was shown, and the experimenter commended the child. The duration of this computer-based test was about 5-10 min.

**Results (repeated-measures ANOVA) for the maladaptive strategies and other strategies of the FEEL-KJ between the three groups (mindfulness-plus, mindfulness, control) and between pre- and posttest**

For the maladaptive strategies, there was only a significant effect of time and interaction effect time x group for self-deprecation (all *p* < 0.05). For self-deprecation there was no difference in the pre-test between groups, *F*(2, 88) = 0.66, *p* = 0.522, η_p_^2^ = 0.015 and in the post-test, *F*(2, 87) = 2.31, *p* = 0.105, η_p_^2^ = 0.050. For the other strategies, the social support subscale had a significant effect on time (*p* < 0.05) and interaction effect on time x group (*p* <0 .001). For social support there was no difference in the pre-test between groups, *F*(2, 88) = 1.19, *p* = 0.522, η_p_^2^ = 0.308 and in the post-test, *F*(2, 87) = 2.42, *p* = 0.095, η_p_^2^ = 0.053. All other subscales did not reach significance. There was no analysis for the subscale emotion control because the internal consistency was too low.

**Table S1**

*Repeated-measures ANOVAs for differences between the three groups (mindfulness-plus, mindfulness, control) in the total score and the ten subscales of the FRKJ 8-16*

| Dependent Variable | Main and interaction effects | | *n* | | *F*(*df*_eff_,*df*_err_) | *p* | η_p_^2^ |
| --- | --- | --- | --- | --- | --- | --- | --- |
| FRKJ 8-16 – Total 88 | | | | |  |  |  |
|  | | Effect of time |  | | 6.93 (1,85) | <0.05 | 0.075 |
| EMP | | Effect of group  Time x group | 90 | | 0.08 (2,85)  2.33 (2,85) | 0.920  0.104 | 0.002  0.052 |
|  | | Effect of time |  | | 8.29 (1,87) | <0.05 | 0.087 |
|  | | Effect of group  Time x group |  | | 0.04 (2,87)  1.54 (2,87) | 0.961  0.221 | 0.001  0.034 |
| S-EF 90 | | | | |  |  |  |
|  | | Effect of time |  | | 6.03 (1,87) | <0.05 | 0.065 |
|  | | Effect of group  Time x group |  | | 0.13 (2,87)  0.24 (2,87) | 0.875  0.784 | 0.003  0.006 |
| S-ES 90 | | | | |  |  |  |
|  | | Effect of time |  | | 0.75 (1,87) | 0.390 | 0.009 |
| SOC | | Effect of group  Time x group | 90 | | 0.66 (2,87)  3.08 (2,87) | 0.521  0.051 | 0.015  0.066 |
|  | | Effect of time |  | | 6.85 (1,87) | <0.05 | 0.073 |
|  | | Effect of group  Time x group |  | | 0.07 (2,87)  2.73 (2,87) | 0.937  0.071 | 0.001  0.059 |
| OPT 90 | | | | |  |  |  |
|  | | Effect of time |  | | 5.09 (1,87) | <0.05 | 0.055 |
|  | | Effect of group  Time x group |  | | 1.05 (2,87)  0.69 (2,87) | 0.354  0.503 | 0.024  0.016 |
| S-C | | Effect of time | 90 | | 5.41 (1,87) | <0.05 | 0.059 |
|  | | Effect of group  Time x group |  | | 1.74 (2,87)  0.74 (2,87) | 0.182  0.479 | 0.038  0.017 |
| PS | |  | 88 | |  |  |  |
|  | | Effect of time |  | | 0.20 (1,85) | 0.657 | 0.002 |
| APS | | Effect of group  Time x group | 88 | | 3.50 (2,85)  4.89 (2,85) | <0.05  <0.05 | .076  .103 |
|  | | Effect of time |  | | 2.81 (1,85) | 0.097 | 0.032 |
| PGI | | Effect of group  Time x group  Effect of time | 88 | | 1.15 (2,85)  0.93 (2,85)  9.22 (1,85) | 0.322  0.400  <0.05 | 0.026  0.021  .098 |
|  | | Effect of group |  | | 0.32 (2,85) | 0.729 | .007 |
|  | | Time x group |  | | 0.42 (2,85) | 0.659 | .010 |
| SI | |  | 88 | |  |  |  |
|  | | Effect of time  Effect of group  Time x group |  | | 0.05 (1,85)  0.71 (2,85)  0.32 (2,85) | 0.827  0.497  0.730 | .001  .016  .007 |
|  | |  | |  |  |  |  |

*Note*. EMP, empathy and perspective-talking skills; S-EF, self-efficacy; S-ES, self-esteem; SOC, sense of coherence; OPT, optimism; S-C, self-control; PS, parental support; APS, authoritative parenting style; PGI, peer group integration; SI, school integration
